# Public Opinion Study on School Health Education Programs: Family Needs Assessment Study †

**DOI:** 10.3390/ijerph22071088

**Published:** 2025-07-08

**Authors:** Hacer Efe, Ünsal Umdu Topsakal

**Affiliations:** Department of Mathematics and Science Education, Education Faculty, Yildiz Technical University, 34220 Istanbul, Turkey; topsakal@yildiz.edu.tr

**Keywords:** public opinion, health education, school, family, needs assessment

## Abstract

Health education programs are important interventions aimed at the acquisition of health knowledge and behaviors that are necessary throughout the lives of individuals of all ages. Considering the importance of health education in structuring the health of the society, it is very important that health education responds to the needs of society and meets its expectations. At this point, public health is protected by providing access to families and communities through health education. In this context, a needs analysis study was conducted with families (students and parents) to establish a health education framework. The study group consisted of 289 volunteer primary and secondary school students and 60 parents who agreed to participate in the study. Semi-structured interviews were conducted with students and parents using a descriptive approach. The needs analysis form prepared by the researchers was used in the interviews and content analysis was applied to the obtained data. The qualitative data obtained from the analyses were interpreted. As a result of the study, it was found that families have expectations and needs in first aid and daily life behaviors (nutrition, hygiene, oral health and diseases) in school health education, and accordingly, school health education can be focused on these specified areas. The fact that families found health knowledge insufficient and considered health education important emphasizes the importance of the knowledge provided by health education and the results. The family health education views obtained in the study can be used in future studies to improve family health behaviors and community health. In addition, family-based school health education can be disseminated with contemporary interventions.

## 1. Introduction

Health is a very important concept that closely concerns every individual in societies globally. Therefore, the quality of health and health education levels of societies are important criteria in determining living standards [1]. Health education is the general name for health interventions carried out to provide individuals with the necessary health information and permanent health behaviors throughout their lives. At this point, school health education is accepted as a valuable field of education by researchers [2]. In fact, the World Health Organization (WHO) has initiated the movement to develop healthy schools within the scope of “schools that continuously strengthen learning and healthy living capacities” and has drawn attention to school health education [3].

While individuals protect their health with the education taught in schools, individuals who protect their health also receive better education. The interdisciplinary structure of school health education aims to deliver health and education in every aspect [4]. In this way, health curriculum programs that include health knowledge, skills, and behaviors are being developed for schools. School health education provides continuous communication and interaction with students and parents. In health education based on the principle of “health for all”, the promotion and development of health is seen as a basic strategy to achieve this principle. In this way, health education programs and studies developed by societies are supported by education grants [5]. Thus, the health literacy of societies increases, and public health develops positively.

**Figure 1:**
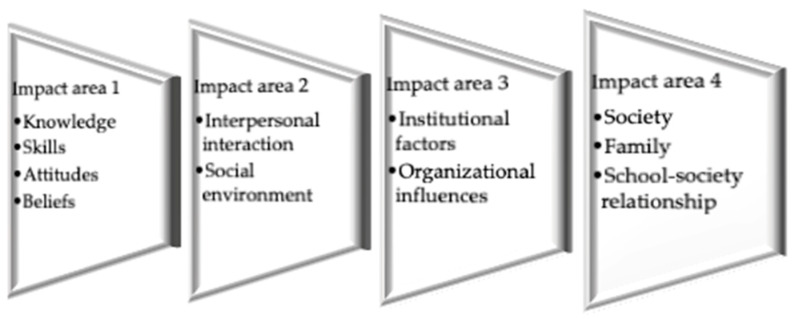
School health education effect levels [10].

In order to reconstruct the deteriorated public health in recent years, it is necessary to instill the idea of a healthy lifestyle in individuals in modern life [6]. The most important educational institutions that undertake this role in modern life are schools, which develop school-based health education programs [7]. School-based health education programs aim to instill a healthy lifestyle in children and their families and to educate them on this subject [8]. Public health can be achieved through the triangle of school, family, and children. In this case, the role of school health education in the construction of a healthy society is undeniable [9]. School health education aims to create healthy living behaviors in individuals and therefore in societies. Accordingly, the impact areas and sub-areas of school health education are given in Figure 1.

School health education programs include educational interventions necessary for public health [11]. In a study conducted by [12], a nutrition-focused health education program was provided to middle school students, and its positive effects on students’ daily nutritional preferences were examined. Another study conducted by [13] found that school health programs were effective in environmental and nutritional issues. Another study conducted by [14] addressed the difficulties of poor coordination, limited educational conditions, and a lack of resources in education, despite the positive effects of school health and nutrition programs in improving students’ health and raising public awareness. The study conducted by [15] emphasized the importance of student participation in developing a common understanding in school health education. Another study conducted by [16] on school health education with parents concluded that sexual health education should be provided in schools on basic topics from an early age. Other studies on school health education can also be found in the literature [17,18,19].

As can be seen, school health education programs are health interventions that closely concern all individuals and communities in society. When it is considered that the most important core community of society is the family, the perspectives of families on health education become quite important. Thus, if the families’ beliefs about health education are determined, it is more possible to adapt health education programs to families and therefore to society. Similarly, the more these programs address the health of a society, the more the health of that society will improve. This interaction emphasizes the importance of school health education programs. In this study, the health belief model, one of the health behavior models, was adopted and examined. The Health Belief Model (HBM) is a health behavior model that evaluates the effects of beliefs and attitudes that protect and affect individuals’ health behaviors. According to this model, the place of beliefs in coping with health problems cannot be denied. The health belief model states that when individuals’ beliefs and attitudes are defined, they will be determined more appropriately for individuals [20]. In this way, this study aims to determine the beliefs of families in school health education (and their needs and expectations) so that school health education appeals more to families and therefore to society. In this context, the opinions of the parties were taken and examined from an analytical perspective. Accordingly, the research problem of the study was determined as “How can the public’s opinions be obtained with the participation of families in the needs analysis for school health education?”.

## 2. Materials and Methods

### 2.1. Study Model

The purpose of this study is to investigate ways to improve and develop school health education. In this way, the descriptive approach was adopted in the study to provide insight into the views of students and parents (families) on health education for health education programs. Descriptive studies are used to gain a general perspective on any research topic or to define a situation more clearly [21]. In the needs assessment conducted in this study, an analysis was conducted focusing on defining the participants’ competencies, expectations, and needs [22]. In the descriptive approach, a needs assessment was conducted in order to collect in-depth information about the current situation and desired goals and to determine learning needs. Needs assessments include techniques for assessing current needs related to an issue or situation. In educational programs, a needs assessment focuses on the learning needs of students, defines them, transforms them into learning tools, and thus forms the basis for the development of teaching materials, activities, tests, or program evaluation strategies. It provides a clear definition of needs, deficiencies, and requests. Requirements represent what the learner knows to function effectively in the target situation. Gaps represent the gap between current and target competencies [23].

### 2.2. Study Group

In selecting the study group, firstly the schools close to the researchers were researched; then the students and parents in the selected school were included in the study. The definite population was preferred for the sample size, and because the qualitative method was preferred in the study, interviews were conducted with the participants, and the answers received from each participant served as a qualitative study.

The study group consists of primary and secondary school students and their parents who live and study in a public school located in an urban area in Turkey. The parents of all students were invited to participate in the study and were asked to participate in this study; however, not all parents were able to participate in the study (family reasons, reluctance, or ineligibility). Accordingly, 289 students participated in the study, while 60 parents were able to participate.

Details of the students who participated in the study are given in Table 1.

Details of the parents who participated in the study are given in Table 2.

In the study, the easily accessible (convenience) sampling method was used in the sample selection. This sampling method aims to provide ease of access for the researcher(s) and to provide speed and practicality to the study [24]. In this context, volunteer students and their parents studying in a public primary or secondary school that provides easy access for the researchers were included in the study. In order to increase the generalizability of the study (in terms of representing a wider population), students from various age groups and grade levels were selected. The parents of the students participating in the study were invited, but since not all parents volunteered for the study, only some of the parents were included in the study. Therefore, the number of parents was more limited than the students. University ethics committee approval and school administrative permission were obtained for the study. In addition, verbal and written consent was obtained from the participants.

### 2.3. Data Collection Tools and Analysis

The data obtained in the study was obtained during the 2022–2023 academic year. The interviews conducted within the scope of the study lasted approximately 5 weeks. The interviews were conducted during school hours convenient for students and parents.

In the study, a “needs analysis interview form” was prepared by the researchers as a data collection tool. A review of the literature was conducted in the development of the interview form. As in other needs analysis studies encountered in the literature, it was decided to include exploratory questions on how to improve health education in this study [25,26]. Accordingly, while developing the questions for the interview form in the study, the aim was to investigate the participants’ views on a possible health education. In this way, the prepared form included questions on the participants’ expectations from health education, the areas needed or undesirable in health education, the participants’ health knowledge competency levels, and the importance of health education. The semi-structured interview form was applied to the students and parents face to face.

The steps followed in the data collection process in the study are as follows:

Literature research: Health education needs analysis studies and health behavior models in the literature were examined.

Creation and development of the data collection tool: At this stage, the development process steps of the data collection tool for the study are given in Table 3.

The needs analysis interview form includes questions regarding the participants’ expectations and needs in health education, health knowledge, and participant beliefs about the importance of health education.

Application of the data collection tool: The developed data collection tool was applied to the participants (students and parents) in the interviews. The data obtained during the interviews were noted by the researchers.

Content analysis was applied in the analysis of the data obtained from the study. In the content analysis process, qualitative data are divided into meaningful parts by the researchers. The codes of these parts are converted into categories and themes, respectively. The interpretation of the data is also carried out together with the analysis [27]. In this study, the content analysis procedure was also applied, and the qualitative data obtained within this scope were examined and first converted into codes, then into categories and themes if necessary. The inductive approach was preferred in the content analysis applied in the study. The reason for this is that the exploratory data collection and analysis process is included in the inductive content analysis. In this content analysis approach, data is examined in depth, and a detailed perspective is attempted to be obtained [28].

In order to ensure validity and reliability in the study, expert opinion was sought in the development of data collection tools. Sufficient time was given to each participant to express their opinions during the interviews. The data obtained from the interviews were examined separately by the researchers, brought together, and compared. Cohen’s Kappa analysis method was preferred in capturing the comparative agreement and the Kappa coefficient was found to be 0.83.

## 3. Results

The answers received from students and parents to the questions were converted into separate codes and categories for each question and presented. The results obtained from students and parents are examined under separate headings.

### 3.1. Results Obtained from Students’ Needs Analysis Interviews

In this title, the results obtained from the interviews with the students are presented separately for the relevant question.

Theme 1: School health education expectations areas

Question: “What are your expectations from health education? Please explain.”

When the answers received from the students for this question were examined, the accident intervention, first aid techniques, and life-saving intervention codes created with the student answers formed the first aid category.

Sample student answers evaluated in this category are as follows:

“I don’t know how to help if I see an accident. It would be good to learn this with health education.” Respondent 1, 3 October 2022, at 09.00 am

“I want to learn how to do first aid, if there are any special techniques. ” Respondent 3, 3 October 2022, at 10.00 am

“For example, how can we save lives by performing first aid, what should we pay attention to when performing first aid.” Respondent 27, 5 October 2022, at 13.30 pm

In the daily life behaviors category, exercise, diseases, oral-dental health, and nutrition codes were reached. Sample student answers evaluated in this category are as follows:

“For example, health education can be given on how to do sports in daily life, what is its importance, because people live very sedentary lives.” Respondent 45, 9 October 2022, at 11.30 am

“I think we can learn how to prevent getting sick, because we get sick very often.” Respondent 139, 17 October 2022, at 14.45 pm

“I think health education that prevents our teeth from decaying can help us learn how to protect our dental health.” Respondent 238, 1 November 2022, at 15.25 pm

A summary of the codes and categories created for students’ health education expectations are provided in Table 4.

Theme 2: School health education needs areas

Question: “What are the health-related topics or health concepts needed in health education?”

Student response examples and corresponding codes in the first aid category are as follows:

“If someone’s heart stops, I think everyone should learn how to massage them.” (Code: heart massage) Respondent 5, 3 October 2022, at 11.00 am

“What can be done or how to give artificial respiration to someone who can’t breathe, I think this is needed.” (Code: artificial respiration) Respondent 53, 10 October 2022, at 09.45 am

Student response examples and corresponding codes in the daily life category are as follows:

“For example; we can get training on how to do our personal hygiene or how to keep our classroom clean.” (Code: cleaning/hygiene) Respondent 81, 12 October 2022, at 13.45 pm

“I think we may need nutrition training on how to eat, how many meals or what to eat at meals, what to buy from the canteen.” (Code: nutrition) Respondent 121, 16 October 2022, at 15.05 pm

“We should get training on the habit of brushing our teeth regularly, why do we go to the dentist, what is the importance of this.” (Code: dental health) Respondent 148, 18 October 2022, at 14.15 pm

“Which medicine does what, why should we take the medicine, health training on this would be good.” (Code: drug information) Respondent 252, 3 November 2022, at 11.45 pm

A summary of the codes and categories created for students’ health education needs are provided in Table 5.

Theme 3: Current health knowledge

Question: “Do you consider your health knowledge enough? Why?”

The majority of students stated that their current health knowledge was not sufficient. As a reason for this, the majority of students stated that the health education in schools was inadequate and that they did not know first aid. Other students did not state any reason.

Theme 4: Importance of school health education

Question: “Do you consider health education important? Why?”

All of the students stated that they considered health education important because it was of vital importance.

Theme 5: School health education topics

Question: “Are there any subjects that you would like not to be included in health education? If so, what are they?”

Students generally stated that there was no topic to be excluded for this question.

### 3.2. Results Obtained from Parents’ Needs Analysis Interviews

In this title, the results obtained from the interviews with the parents are presented separately for the relevant question.

Theme 1: School health education expectations areas

Question: “What are your expectations from health education? Please explain.”

The parents’ answers were analyzed, and a daily life behaviors category was created. The codes in the daily life behaviors category include nutrition, diseases, exercise, cleaning, and dental health. Sample answers from parents are as follows:

“Health education on the importance of walking and doing sports in our daily lives can be useful.” Respondent 67, 11 October 2022, at 11.00 am

“I think individuals should be taught personal daily and monthly cleaning behaviors, cleaning starts in schools.” Respondent 94, 13 October 2022, at 10.00 am

“I can recommend health education on how to take care of our teeth, children’s teeth are usually rotten, so brushing teeth should be taught.” Respondent 116, 16 October 2022, at 13.15 pm

“Health education can be conducted on what to do to avoid getting sick.” Respondent 129, 17 October 2022, at 10.50 am

“The importance of balanced nutrition can be emphasized in health education, information about not skipping meals and the content of meals can be included.” Respondent 157, 19 October 2022, at 14.45 pm

A summary of the codes and categories created for parents’ health education expectations are provided in Table 6.

Theme 2: School health education needs areas

Question: “What are the health-related topics or health concepts needed in health education?”

The parents’ responses were analyzed.

The first aid category includes the importance of first aid and the application steps codes. Sample student responses evaluated in this category are as follows:

“Health education should teach how to apply first aid in situations that require it.” Respondent 34, 6 October 2022, at 11.45 am

“First aid is an area that everyone should learn, its importance should be taught with health education.” Respondent 73, 12 October 2022, at 12.00 am

“First aid techniques should be taught in health education, we should learn which technique to apply in which situation.” Respondent 107, 16 October 2022, at 09.15 am

The daily healthy living knowledge category includes the codes of cleanliness, proper nutrition, and health protection. Examples of answers evaluated in these categories are as follows:

“Health education should teach cleanliness to every individual in order to be clean and hygienic as a society.” Respondent 213, 30 October 2022, at 11.45 am

“What to pay attention to in nutrition, what to eat or not to eat, a nutrition education regarding these can be included in health education.” Respondent 233, 1 November 2022, at 13.45 pm

“Health education should address ways to protect ourselves from diseases and preserve our health.” Respondent 247, 3 November 2022, at 10.00 am

“I think health education should include the importance and content of proper nutrition against obesity.” Respondent 265, 6 November 2022, at 11.00 am

“In my opinion, health education can teach us how to maintain our health in general and what we can do for this.” Respondent 283, 9 November 2022, at 14.25 pm

“How we can keep our bodies and the environment we live in clean and what its importance is should be included in health education.” Respondent 285, 9 November 2022, at 15.15 pm

A summary of the codes and categories created for parents’ health education needs are provided in Table 7.

Theme 3: Current health knowledge

Question: “Do you consider your health knowledge enough? Why?”

The majority of parents found their current health information to be inadequate due to current developments in health and the fact that they did not receive comprehensive health education.

Theme 4: Importance of school health education

Question: “Do you consider health education important? Why?”

The majority of parents found health education important because health is vital and improves our quality of life.

Theme 5: School health education topics

Question: “Are there any subjects that you would like not to be included in health education? If so, what are they?”

Parents generally stated that there was no topic to be excluded in this question.

## 4. Discussion

### 4.1. Discussion on the Needs and Expectations of Health Education

When the results obtained on expectations and needs in the study were examined, it was found that both students and parents needed school health education in the areas of first aid and daily life behaviors (nutrition, exercise, dental health, and diseases). In a similar study [29], students stated that they needed awareness in daily life behaviors (nutrition, hygiene, diseases, etc.) in health education. In a parallel study [30], students and parents reached the conclusion that there was a need in daily life behaviors such as nutrition and sports in the needs assessment of health education. In addition, in terms of expectations from school health education, it was found that students had expectations from first aid and daily life behaviors, and parents had expectations from school health education in the areas of daily life behaviors. Similarly, in the study conducted by [31] on the needs assessment, participants stated that health education should be developed on healthy daily behaviors.

### 4.2. Discussion on the Importance and Necessity of Health Education

When the results on the importance and necessity of health education are examined, it is seen that students and parents generally see health education as important and current health information as insufficient. In a study conducted by [32], the participants stated that they have little (limited) health information; they can obtain health information through health education. They also find health education important because health is of vital importance. Similarly, in the health needs analysis study conducted by [33], the participants stated that health education is an important issue. Parents find health information insufficient due to current developments in the field of health and their inability to receive comprehensive health education. Similarly, in the needs analysis conducted by [34], the participants stated that they want to receive health education. Another study [35] stated that the participants have insufficient health information and that health education should be expanded. In addition, the parents stated that they find health education important because health is of vital importance and increases the quality of life. In a similar study conducted by [36], participants highlighted the importance of health school education. 

Since the data for this study was collected in the 2023 academic year, it may be limited in reflecting current results. In addition, the study is limited to the study population that includes certain age groups and qualitative data collection techniques and tools. In order to generalize the study, studies can be conducted with various participants and different data collection tools. Finally, social desirability bias was not detected in the study.

## 5. Conclusions

In this study, the importance of individuals’ health beliefs and attitudes in creating healthy behaviors in individuals, and therefore in society, in line with the adopted health belief model, was taken as the basis. In this way, a needs assessment was conducted with families in order to conduct a public opinion survey on school health education programs. In the assessment, an attempt was made to determine the attitudes, expectations, and needs of families towards school health education programs. It is expected that the results of the study will contribute to the development of school health education programs and thus to public health. According to the results of the study, school health education programs can be developed and disseminated.

Future health education studies can focus on certain areas based on the results of this study. School health education can be made more comprehensive and widespread in order to maintain and improve family health.

## Figures and Tables

**Table 1:** The characteristics of the students (*n* = 289).

Ages	Girl *n* (%)	Boy *n* (%)
10–12 age	55 (34.5%)	104 (65.5%)
13–14 age	71 (54.6%)	59 (45.4%)
Total	126 (43.6%)	163 (56.4%)

**Table 2:** The characteristics of the parents (*n* = 60).

Education Level	Female *n* (%)	Male *n* (%)
High school	21 (35%)	3 (5%)
Undergraduate	12 (30%)	24 (40%)
Total	33 (55%)	27 (45%)

**Table 3:** Steps of the development of the data collection.

Steps	Content
Step 1	Research of the literature data collection tools in the adopted model (health belief model)
Step 2	Researchers’ decision on the interview form
Step 3	Determining the points to be examined in the interview form (expectation, need, and belief)
Step 4	Researchers’ creation of a question pool for the interview form
Step 5	Submission of the created question pool to expert opinion
Step 6	Elimination and arrangement of questions in line with expert opinion
Step 7	Development questions and finalization

**Table 4:** Codes and categories generated from students’ expectations.

Categories	Codes	Frequency *n* (%)
Daily life behaviors	Daily life situations	106 (37%)
	Diseases prevention	86 (30%)
	Nutrition	9 (3%)
	Oral-dental health	9 (3%)
First aid	First aid techniques	68 (23%)
	Accident intervention	5 (2%)
	Life-saving intervention	5 (2%)

**Table 5:** Codes and categories generated from students’ needs.

Categories	Codes	Frequency *n* (%)
Daily life behaviors	Diseases prevention	128 (44%)
	Drug information	23 (8%)
	Nutrition	10 (3%)
	Dental health	6 (2%)
First aid	Heart massage	15 (5%)
	Artificial respiration	13 (4%)
	Intervention examples	7 (2%)

**Table 6:** Codes and categories generated from parents’ expectations.

Categories	Codes	Frequency *n* (%)
Daily life behaviors	Nutrition	17 (6%)
	Diseases prevention	13 (4%)
	Exercise	9 (3%)
	Cleaning	6 (2%)
	Dental health	6 (2%)

**Table 7:** Codes and categories generated from parents’ needs.

Categories	Codes	Frequency *n* (%)
First aid	Application steps	18 (6%)
	Definition	16 (5%)
Daily life behaviors	Infectious diseases prevention	12 (4%)
	Cleanliness	10 (3%)
	Proper nutrition	5 (2%)

## Data Availability

Data presented in this study are available upon request from the authors (H.E., Ü.U.T.). Data are not publicly available due to privacy concerns.
